# Efficacy of different types of aerobic exercise in fibromyalgia syndrome: a systematic review and meta-analysis of randomised controlled trials

**DOI:** 10.1186/ar3002

**Published:** 2010-05-10

**Authors:** Winfried Häuser, Petra Klose, Jost Langhorst, Babak Moradi, Mario Steinbach, Marcus Schiltenwolf, Angela Busch

**Affiliations:** 1 Department of Internal Medicine I, Klinikum Saarbrücken, Winterberg 1, D-66119 Saarbrücken, Germany; 2 Department of Psychosomatic Medicine, Technische Universität München, Langestr. 3, D-81675 München, Germany; 3 Department of Internal Medicine V (Integrative Medicine), University of Duisburg-Essen, Kliniken Essen-Mitte, Am Deimelsberg 34a, D-45276 Essen, Germany; 4 Orthopaedic Clinic, University of Heidelberg, Schlierbacher Landstraße 200, D-69118 Heidelberg, Germany; 5 School of Physical Therapy, University of Saskatchewan, Saskatoon, 1121 College Drive, Saskatoon SK S7N OW3, Canada

## Abstract

**Introduction:**

The efficacy and the optimal type and volume of aerobic exercise (AE) in fibromyalgia syndrome (FMS) are not established. We therefore assessed the efficacy of different types and volumes of AE in FMS.

**Methods:**

The Cochrane Library, EMBASE, MEDLINE, PsychInfo and SPORTDISCUS (through April 2009) and the reference sections of original studies and systematic reviews on AE in FMS were systematically reviewed. Randomised controlled trials (RCTs) of AE compared with controls (treatment as usual, attention placebo, active therapy) and head-to-head comparisons of different types of AE were included. Two authors independently extracted articles using predefined data fields, including study quality indicators.

**Results:**

Twenty-eight RCTs comparing AE with controls and seven RCTs comparing different types of AE with a total of 2,494 patients were reviewed. Effects were summarised using standardised mean differences (95% confidence intervals) by random effect models. AE reduced pain (-0.31 (-0.46, -0.17); *P *< 0.001), fatigue (-0.22 (-0.38, -0.05); *P *= 0.009), depressed mood (-0.32 (-0.53, -0.12); *P *= 0.002) and limitations of health-related quality of life (HRQOL) (-0.40 (-0.60, -0.20); *P *< 0.001), and improved physical fitness (0.65 (0.38, 0.95); *P *< 0.001), post treatment. Pain was significantly reduced post treatment by land-based and water-based AE, exercises with slight to moderate intensity and frequency of two or three times per week. Positive effects on depressed mood, HRQOL and physical fitness could be maintained at follow-up. Continuing exercise was associated with positive outcomes at follow-up. Risks of bias analyses did not change the robustness of the results. Few studies reported a detailed exercise protocol, thus limiting subgroup analyses of different types of exercise.

**Conclusions:**

An aerobic exercise programme for FMS patients should consist of land-based or water-based exercises with slight to moderate intensity two or three times per week for at least 4 weeks. The patient should be motivated to continue exercise after participating in an exercise programme.

## Introduction

The key symptoms of fibromyalgia syndrome (FMS) are chronic widespread (both sides, above and below waist line, and axial skeletal) pain, fatigue, sleep disturbances and tenderness on palpation [1]. The estimated prevalence of FMS in western countries ranges from 2.2 to 6.6% [2]. Comorbidities with other functional somatic syndromes and mental disorders are common [3]. FMS is associated with high utilisation and costs of health services. Effective treatment options are therefore needed for medical and economic reasons [4].

Systematic reviews and evidence-based guidelines provide healthcare professionals and patients with a guide through the great variety of pharmacological and nonpharmacological treatment options in FMS. Three evidence-based guidelines available on the management gave different grades of recommendation for aerobic exercises (AE) (aerobic exercise with and without additional strength and flexibility training) in FMS. The American Pain Society [5] and the guidelines of the Association of the Scientific Medical Societies in Germany [6] gave the highest grade of recommendation for AE. The European League Against Rheumatism judged the published evidence for the efficacy of AE to be lacking [7]. Qualitative reviews on the efficacy of AE in FMS that searched the literature until December 2006 came to different conclusions on the short-term and long-term efficacy of AE in FMS [8-10].

More recently, Jones and Lipton reviewed over 70 FMS exercise studies and found similar results when protocols included yoga, tai chi and other movement-based therapies [11]. Two meta-analyses on exercise in FMS have been conducted. Busch and colleagues searched the literature until July 2005. Owing to significant clinical heterogeneity among the studies, only six studies with AE were meta-analysed. Moderate quality evidence was found that AE had positive effects on global well-being and physical function, but not on pain at post treatment [12]. The Ottawa Panel searched the literature until December 2006 and found most improvements for pain relief and increase of endurance at post treatment [13]. Outcomes at follow-up were not meta-analysed.

Not only the question of efficacy but also that of the dose and type of AE need to be clarified. The American Pain Society recommended encouraging patients to perform moderately intense AE (60 to 70% of age-adjusted predicted maximum heart rate (maxHR)) two or three times per week [5]. The evidence of this recommendation has not been tested by meta-analyses of head-to-head comparisons of different types and volumes of AE. Moreover, the question of whether continuing AE is required to maintain a symptom reduction had not been systematically addressed.

The aims of the present systematic review were to update the literature on AE in FMS and to assess whether AE has beneficial effects at post treatment and at follow-up on the key domains of FMS (pain, sleep, fatigue, depressed mood), compared with other therapies. In contrast to the Cochrane review [12], we intended to meta-analyse the outcomes of all randomised controlled trials (RCTs) available. Another aim was to asses which types, volumes and intensities of AE are effective by performing head-to-head comparisons of RCTs with different types and intensities of AE. The final aim was to assess whether ongoing exercise is necessary to maintain potential positive effects of AE.

## Materials and methods

The present review was performed according to the Preferred Reporting Items for Systematic Reviews and Meta-Analyses statement [14] and the recommendations of the Cochrane Collaboration [15].

### Protocol

Methods of analysis and inclusion criteria were specified in advance. We used the review protocol of our systematic review on multicomponent therapy in FMS [16].

### Eligibility criteria

#### Types of studies

A RCT design comparing AE with a control group receiving no treatment, treatment as usual, attention control or any pharmacological or nonpharmacological therapy, or with head-to-head comparisons of different types or intensities of AE were included. Studies without randomisation were excluded.

#### Types of participants

Patients of any age diagnosed with FMS on recognised criteria were included.

#### Types of intervention

AE was assumed if the reported target heart rate of the training protocol was at least (on average) 40% of maxHR or if the training protocol included exercise involving at least one-sixth of the skeletal muscles (for example, walking, running, biking, aerobics, vibrations). At least 50% of the training session should consist of AE. In the case of mixed exercise, defined as a combination of AE with stretching and/or muscle strength [17], the length of AE should exceed the time with other types of exercise. Stretching during warm-up and cool-down periods was not defined as mixed exercise. No restrictions on frequency or duration of training were made.

We excluded studies or study arms in which AE was part of multicomponent therapy defined as a combination of AE with psychological therapy (structured education or relaxation therapy, cognitive-behavioural therapy) [16]. We excluded studies or study arms with balneotherapy (warm-water treatment without exercise).

#### Types of outcomes measures

Studies should assess at least one key domain of FMS (pain, sleep, fatigue, depressed mood and health-related quality of life (HRQOL)) (primary outcome measures). Secondary outcome measures were any measure of physical fitness.

### Data sources and searches

The electronic bibliographic databases screened included the Cochrane Central Register of Controlled Trials (CENTRAL), EMBASE, MEDLINE, PsychInfo and SPORTDISCUS (through 31 March 2009). The search strategy for MEDLINE is detailed in Additional file 1. The search strategy was adapted for each database as necessary. No language restrictions were made. Only fully published papers were reviewed. In addition, reference sections of original studies, systematic reviews [8-10] and evidence-based guidelines on the management of FMS [4-6] were screened manually.

### Study selection

The search was conducted by two authors (PK, JL). Two authors screened the titles and the abstracts of potentially eligible studies identified by the search strategy detailed above independently (PK, JL). The full-text articles were then examined independently by two authors to determine whether they met the selection criteria (MSc, JL). Discrepancies were rechecked and consensus was achieved by discussion. If needed, two other authors reviewed the data to reach a consensus (AB, WH).

### Data collection process

Two authors independently extracted the data using standard extraction forms [16] (BM, MSc). Discrepancies were rechecked and consensus was achieved by discussion. If needed, a third author reviewed the data to reach a consensus (WH).

Based on our experiences of former systematic reviews in which none of the contacted authors provided these details on request, we did not ask for clarifications of study design in case of unclear randomisation, blinding or concealment of treatment allocation. We searched for further details of the study design in a Cochrane review [12].

When means or standard deviations (SDs) were missing, attempts were made to obtain these data through contacting 12 trial authors. Additional data were provided by four authors (see Tables 1 and 2). Where SDs were not available from the trial authors, they were calculated from *t *values, confidence intervals or standard errors when reported in articles [15]. If only the median was given, the median was used instead of the mean and a SD was substituted that was calculated as the mean of the SDs available for studies that used the same outcome scale.

**Table 1:** Risk of bias (internal and external validity) of the randomised controlled trials' analysis

Author, year	Adequate randomisation	Adequate allocation concealment	Blinding of assessor	Intention-to-treat analysis	Inclusion of patients with mental disorders
Alentorn, 2008	0	0	+	-	+
Altan, 2004	0	0	0	-	-
Assis, 2006	+	+	-	+	-
Bircan, 2006	0	0	0	-	+
Buckelew, 2008	0	0	0	-	+
Da Costa, 2005	+	+	+	+	+
Ecvik, 2008	0	0	0	-	+
Etnier, 2009	0	0	0	-	+
Fontaine, 2007	0	0	0	-	+
Gowans, 2001	0	0	0	-	+
Gusi, 2006	0	0	0	-	-
Jentoft, 2001	0	0	-	+	+
Jones, 2008	+	+	+	+	-
King, 2002	+	0	+	+	+
Martin, 1996	0	0	+	+	+
McCain, 1988	0	0	+	-	+
Mengshoel, 1992	+	0	0	+	+
Meyer, 2000	0	0	0	-	+
Munguia, 2008	+	+	+	+	-
Nichols, 1994	0	0	0	-	+
Noregaard, 1997	0	0	0	+	+
Ramsay, 2000	0	0	+	+	0
Redondo, 2004	+	0	0	+	-
Richards, 2002	+	+	+	+	+
Rooks, 2007	+	+	+	+	0
Schachter, 2003	+	+	+	+	+
Sencan, 2004	0	0	0	+	0
Stephens, 2008	+	+	+	+	+
Tomas-Carus, 2008	0	+	+	+	0
Valim, 2003	0	0	+	+	+
Valkeinen, 2008	+	0	0	+	0
Van Santen, 2001	0	-	-	-	0
Van Santen, 2002	0	0	+	+	-
Vitorino, 2006	+	+	-	+	-
Wigers, 1996	0	0	+	+	0

+, yes; 0, unclear; -, no.

**Table 2:** Effect sizes of aerobic and mixed exercise on selected outcome variables

Outcome title	Number of study arms	Number of patients on aerobic exercise	Effect size^a^	Test for overall effect *P *value	Heterogeneity, *I*^2^; τ^2 ^(%)
Post treatment					
01 Pain	29	567	-0.31 (-0.46, -0.17)	<0.001	26; 0.03
02 Fatigue	16	364	-0.22 (-0.38, -0.05)	0.009	9; 0.01
03 Sleep	9	184	0.01 (-0.19, 0.21)	0.92	0; 0
04 Depressed mood	19	456	-0.32 (-0.53, -0.12)	0.002	51; 0.10
05 HRQOL	25	526	-0.40 (-0.60, -0.20)	<0.001	63/0.15
06 Physical fitness	20	339	0.65 (0.38, 0.93)	<0.001	71/0.20
Latest follow-up					
01 Pain	9	187	-0.13 (-0.80, 0.54)	0.08	0/0
02 Fatigue	4	93	-0.23 (-0.62, 0.17)	0.26	42/0.07
03 Sleep	4	84	0.17 (-0.14, 0.47)	0.28	0/0
04 Depressed mood	8	151	-0.44 (-0.88, 0.01)	0.05	71/0.22
05 HRQOL	8	221	-0.27 (-0.48, -0.05)	0.02	14/0.01
06 Physical fitness	5	99	0.65 (0.35, 0.96)	<0.001	0/0

HRQOL: health-related quality of life. ^a^Standardised mean difference (95% confidence interval).

### Data items

The data for the study setting, participants, exclusion criteria, interventions, co-therapies, attendance rates, side effects reported and outcomes sought are presented in Tables 2 and 3.

**Table 3:** Effect sizes of head-to-head comparisons of different types of aerobic exercise on selected outcome variables

Outcome title post treatment	Number of studies	Number of patients	Effect size^a^	Test for overall effect, *P *value	Heterogeneity, *I*^2^; τ^2 ^(%)
Moderate intensity versus low intensity					
01 Pain	2	68	-0.08 (-1.41, 1.26)	0.91	78; 0.96
02 Depressed mood	2	68	-0.16 (-0.67, 0.13)	0.53	0; 0.
03 Physical fitness	2	68	0.25 (-0.26, 0.75)	0.34	0; 0
Land-based versus water based exercise					
01 Pain	9	187	-0.13 (-0.80, 0.54)	0.08	0/0
02 Depressed mood	8	151	-0.44 (-0.88, 0.01)	0.05	71/0.22

^a^Standardised mean difference (95% confidence interval).

When researchers reported more than one measure for an outcome, we used a predefined order of preference for analysis (details available on request).

If studies had two or more potential control groups, we used the following order to select for control group: treatment as usual, attention placebo, and active control to select the control group.

### Risk of bias in individual studies

To ascertain the internal and external validity of the eligible RCTs, two pairs of reviewers (BM, WH; and MSc, Mst) working independently and with adequate reliability determined the adequacy of randomisation, concealment of allocation, blinding of outcome assessors and adequacy of data analysis (was intention-to-treat-analysis performed?) (internal validity). Furthermore we chose the item 'Were patients with mental disorders frequently associated with FMS (depressive and anxiety disorders) included in the studies?' as the marker of external validity.

### Summary measures

Meta-analyses were conducted using RevMan Analyses software (RevMan 5.0.17) from the Cochrane collaboration [18]. Standardised mean differences (SMDs) were calculated by means and SDs or change scores for each intervention. The SMD used in Cochrane reviews is the effect size known as Hedge's (adjusted) *g *[15]. Examination of the combined results was performed by a random effects model (inverse variance method), because this model is more conservative than the fixed effects model and incorporates both within-study and between-study variance [19]. We used Cohen's categories to evaluate the magnitude of the effect size, calculated by the SMD: *g *> 0.2 to 0.5, small effect size; *g *> 0.5 to 0.8, medium effect size; *g *> 0.8, large effect size [20].

### Planned methods of analysis

Heterogeneity was tested using the *I*^2 ^statistic, with *I*^2 ^> 50% indicating strong heterogeneity. τ^2 ^was used to determine how much heterogeneity was explained by subgroup differences [15].

### Risk of bias across studies

Potential publication bias - that is, the association of publication probability with the statistical significance of study results - was investigated using visual assessment of the funnel plot (plots of effect estimates against its standard error) calculated by RevMan Analyses software. Publication bias may lead to asymmetrical funnel plots [15]. Moreover, we checked a potential small sample size bias by a sensitivity analysis of studies with very small (<25), small (25 to 50) and medium (>50) sample sizes.

### Additional analyses

#### Subgroup analysis

The following subgroup analyses were pre-specified: types of AE (land-based, water-based and mixed; AE as monotherapy or combined with flexibility and/or strength), intensity of AE (very low intensity, <50% of maxHR; low intensity, 50 to 60% of maxHR; moderate intensity, 60 to 80% maxHR; intensity left up to patient), frequency of AE per week (1 time/week, 2 times/week, 3 times/week and >3 times/week), duration of the study (<7 weeks, 7 to 12 weeks, >12 weeks) and duration of total aerobic exercise (<1,000 minutes, 1,000 to 2,000 minutes, >2,000 minutes), and type of control group (attention placebo, treatment as usual, other active therapy). These subgroup analyses were also used to examine potential sources of clinical heterogeneity.

#### Sensitivity analyses

The following sensitivity analyses were pre-specified: inadequate or unclear versus adequate sequence generation; inadequate or unclear allocation versus adequate concealment; intention-to-treat analysis, no versus yes; studies that provided medians of outcomes versus means of outcomes; and patients with mental disorders frequently associated with FMS excluded (yes or unclear). These sensitivity analyses were also used to examine potential sources of methodological heterogeneity.

## Results

### Study selection

**Figure 1:**
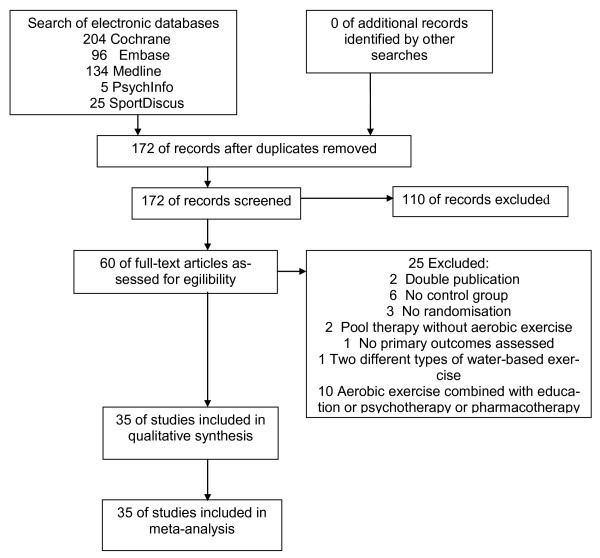
**Preferred Reporting Items for Systematic Reviews and Meta-Analyses flow chart**. Schematic description of the results of the literature search.

The literature search produced 464 citations, of which 292 were double hits (study found in at least two data sources). By screening, 110 records were excluded: 23 evaluated AE, but not in FMS; 19 did not evaluate AE in FMS; 52 were review articles; and 18 were case reports or commentaries. Sixty of the full-text articles assessed for eligibility, and 25 full-text articles were excluded for the following reasons: two for publication of different outcomes of one trial in two publications [21,22]; six for lacking a control group [23-28]; three for lacking randomisation [29-31]; two because one could not conclude from the study protocol that the exercises performed met the predefined criteria of AE [32,33]; one because two different types of water-based exercise with similar intensity were compared [34]; one because the study did not assess a primary outcome measure [35]; and 10 because AE was combined with education or psychotherapy or pharmacotherapy [36-45]. Three RCTs comparing different intensities of AE [46-48], four RCTs comparing land-based with water-based exercise [49-52] and 28 RCTs with 29 study arms comparing AE with controls [53-80] were included in the qualitative and quantitative analyses (see Figure 1).

### Study characteristics

#### Setting, referral and exclusion criteria (representativeness of study samples)

Fourteen studies each were conducted in North America, 13 studies in Europe and four studies each in South America (Brazil) and Asia (Turkey) (see Additional files 1, 2 and 3). Patients were recruited by register of hospitals, referral (general practitioner, rheumatologist, hospital departments), local self-help groups and newspaper advertisement. Thirty-two studies were conducted within the setting of a university, three within district hospitals. All studies were single-centre based. One study had two AE study arms.

Thirty-one studies excluded patients with internal diseases or with orthopaedic diseases precluding AE. Sixteen studies excluded patients with mental disorders including depression. Four studies excluded patients with unresolved litigation. No study reported comorbidities of the patients.

#### Participants

The median of the mean age of the participants was 45 years (13 to 59 years). One study included only children and adolescents. The median of the percentage of women was 100% (71 to 100%).

#### Interventions

AE was supervised by a trainer in 32 studies. AE included cycling, walking, aquatic jogging, games, dance and rhythmic or boxing movements. Aerobic intensity was reported in 27 studies as a target heart rate or percentage age-predicted maxHR determined by standard equations. Percentage maxHRs were usually progressive and ranged from 40 to 80% of the age-predicted maximum. The target heart rate of 21 studies was between low and moderate intensity (50 to 80%). Only one study prescribed a very low intensity (maxHR 30 to 50%), and three studies recommended that patients should exercise with a moderate intensity subjectively determined by the patient without measuring the heart rate. Three studies did not report the recommended intensity.

Sixteen studies reported the attrition rates, with a median of 67% (range 27 to 90%).

In 12 studies the controls received treatment as usual, and in 10 studies they received another active therapy (spa, hot packs, structured education, supervised relaxation, cognitive behavioural therapy, muscle strengthening, stretching). In six studies an attention control was used (nonstructured education, supervised recreational therapies, transcutaneous electrical neurostimulation or pharmacological placebo) (see Additional file 1).

Three studies compared different intensities of land-based AE, and four studies compared water-based AE with land-based AE (see Additional file 2).

A total 694/889 (78.1%) of the patients in the AE groups and 617/742 (83.1%) in the control groups completed therapy (*z *= -0.3, *P *= 0.7).

Fourteen studies performed follow-ups. The median of the latest follow-up was 26 (12 to 208) weeks. Five studies reported that the patients were motivated to continue exercise [51,56,70,71,75]. One study recommended no exercise until follow-up evaluation [61]. Two studies assessed the effects of continuing exercise on outcomes [25,80]. One study compared the outcomes of continuers of exercise versus noncontinuers at follow-up without mentioning whether continuing exercise had been recommended [80]. Two studies performed an uncontrolled follow-up [37,60].

#### Outcomes

There was a great variety of most outcomes measures (see Additional files 1, 2 and 3). Eleven studies reported on side effects. Five studies reported that no side effects occurred, and six studies reported an increase of symptoms leading to a drop out in some cases. Only six patients assigned to AE were designated to have an adverse event possibly related to exercise (metatarsal stress fracture, plantar fasciitis, ischialgia, transient knee pain).

### Risk of bias within studies

Only two studies fulfilled all predefined criteria of internal and external validity (see Table 1).

### Results of individual studies

The means, SDs, sample sizes and effect estimates of each study can be seen in the forest plots (see Additional files 4, 5, 6, 7, 8, 9, 10, 11, 12 and 13).

### Synthesis of results

#### Aerobic exercise patients versus controls

Data are reported as the SMD (95% confidence interval).

At post treatment, AE reduced pain (-0.31 (-0.46, -0.17); *P *< 0.001), fatigue (-0.22 (-0.38, -0.05); *P *= 0.006), depressed mood (-0.32 (-0.53, -0.12); *P *= 0.002) and limitations of HRQOL (-0.40 (-0.60, -0.20); *P *< 0.001), and improved physical fitness (0.65 (0.38, 0.93); *P *< 0.001), compared with controls. The effect on sleep (0.01 (-0.19, 0.21); *P *= 0.92) was not significant. Based on Cohen's categories, the effects were small for pain, fatigue, depression and HRQOL, and were medium for physical fitness (see Table 4).

**Table 4:** Subgroup analysis for the effect size on pain at post treatment

Outcome title	Number of study arms	Number of patients on AE	Effect size^a^	Test for overall effect, *P *value	Heterogeneity, *I*^2^; τ^2 ^(%)
Type of exercise					
Land-based	22	463	-0.29 (-0.46,-0.13)	0.0005	27; 0.03
Water-based	3	61	-0.67 (-1.04,-0.29)	0.0005	0; 0
Mixed	4	43	-0.03 (-0.45,0.39)	0.89	0; 0
Type of exercise					
AE only	12	273	-0.35 (-0.61,-0.09)	0.0008	48; 0.09
AE combined with other exercise	17	294	-0.28 (-0.45,-0.15)	0.001	0; 0
Duration of study					
<7 weeks	2	32	-1.16 (-1.86,-0.48)	0.001	36; 0.09
7 to 12 weeks	13	194	-0.24 (-0.50,-0.02)	0.03	16; 0.02
>12 weeks	12	338	-0.24 (-0.40,-0.08)	0.004	0; 0
Frequency of training/week					
1 time/week	2	37	-0.07 (-0.54,03.9)	0.48	Not applicable
2 times/week	5	127	-0.69 (-0.95,-0.27)	0.0004	35; 0.06
3 times/week	16	241	-0.35 (-0.62,-0.09)	0.009	48; 0.10
>3 times/week	4	142	-0.13 (-0.38, 0.13)	0.33	2; 0
Total duration aerobic exercise^b^					
<1,000 minutes	10	175	-0.47 (-0.86,-0.08)	0.02	62; 0.19
1,000 to 2,000 minutes	9	175	-0.36 (-0.59,-0.13)	0.002	0; 0
>2,000 minutes	8	217	-0.15 (-0.34, 0.05)	0.15	0; 0
Intensity of AE^c^					
<50% maxHR	1	37	-0.09 (-0.54, 0.36)	Not applicable	Not applicable
Left up to patient	2	79	-0.42 (-0.77, -0.07)	0.02	0; 0
> 50% maxHR	21	367	-0.26 (-0.42,-0.11)	0.0007	0; 0
Type of control group					
Attention placebo	7	229	-0.27 (-0.62, 0.07)	0.12	67; 0.12
Therapy as usual	10	147	-0.47 (-0.71,-0.24)	<0.0001	0; 0
Active therapy	10	191	-0.27 (-0.49,-0.06)	0.01	0; 0

AE, aerobic exercise; maxHR, maximum of age-adjusted maximum heart rate. ^a^Standardised mean difference (95% confidence interval). ^b^If no precise duration of AE was given, 50% of the total exercise time was assumed for aerobic exercise. ^c^Studies that did not report the intensity of training were excluded from analysis.

At latest follow-up, AE reduced depressed mood (-0.44 (-0.88, 0.01); *P *= 0.05) and limitations of HRQOL (-0.27 (-0.48, -0.05); *P *= 0.01), and improved physical fitness (0.65 (0.35, 0.96); *P *< 0.001), compared with controls. The effects were small for depressed mood and HRQOL, and were medium for physical fitness. The effects on pain (-0.13 (-0.80, 0.54); *P *= 0.08), fatigue (-0.23 (-0.62, 0.17); *P *= 0.26) and sleep (0.17 (-0.14, 0.47); *P *= 0.26) were not significant (see Table 4).

#### Land-based versus water-based aerobic exercise

There were no significant effects of water-based AE versus land-based AE on the outcomes pain and depressed mood at post treatment (see Table 2).

#### Moderate-intensity versus low-intensity aerobic exercise

There were no significant effects of moderate-intensity compared with low-intensity AE on the outcomes pain, depressed mood and physical fitness at post treatment (see Table 3).

#### Effects of continuing exercise

One study found that continuers of exercise at follow-up reported less pain and depression than those who did not exercise [80]. One study found that exercising at follow-up was related to improvements in physical function and mood [37]. One study reported that pain returned close to the pretraining level during the subsequent de-training [61].

### Risk of bias across studies

There was only substantial heterogeneity in the comparisons of depressed mood and HRQOL at post treatment and for depressed mood at latest follow-up (see Table 2). On visual inspection, the funnel plots of the outcomes post treatment were symmetrical and were thus not indicative for a publication bias (see Additional file 14). Studies with small sample sizes had no significant effect on pain at post treatment (see Table 5).

**Table 5:** Sensitivity analysis for the effect size on pain at post treatment

Outcome title	Number of study arms	Number of patients on aerobic exercise	Effect size^a^	Test for overall effect, *P *value	Heterogeneity, *I*^2^; τ^2 ^(%)
Adequate sequence generation					
Adequate	11	251	-0.20 (-0.38, -0.01)	0.04	0; 0
Unclear or nonadequate	18	348	-0.39 (-0.61, -0.18)	0.0004	39; 0.07
Allocation concealment					
Adequate	8	223	-0.24 (-0.47, -0.01)	0.04	24; 0.02
Unclear or nonadequate	19	344	-0.35 (-0.54, -0.16)	0.0002	28; 0.04
Blinding of assessor					
Yes	12	306	-0.20 (-0.36,-0.03)	0.02	0; 0
No or unclear	15	261	-0.41 (-0.66,-0.16)	0.001	38;0.08
ITT analysis					
Yes	13	315	-0.22 (-0.39, -0.06)	0.009	0; 0
No	14	252	-0.39 (-0.62, -0.16)	0.001	36; 0
Adequacy of outcomes for meta-analysis					
Yes (means)	24	517	-0.35 (-0.51,-0.19)	<0.0001	28;0.04
No (medians)	3	43	-0.05 {-0.43,-0.32)	0.78	0; 0
Sample size					
<25	3	30	-0.33 (-1.00,0.33)	0.33	34; 0.12
25 to 50	15	188	-0.41 (-0.70,-0.13)	0.005	46;0.11
>50	9	349	-0.23 (-0.39,-0.08)	0.004	0; 0
Patients with mental disorders included					
Yes	16	312	-0.43 (-0.78, -0.08)	0.02	73; 0.28
No or unclear	14	315	-0.40 (-0.61, -0.19)	0.0002	38; 0.05

ITT, intention to treat. ^a^Standardised mean difference (95% confidence interval).

### Additional analyses

#### Subgroup analysis

Subgroup analyses according to the types of AE, frequency, total time and intensity of AE and type of control groups did not change the significant effect of AE on pain at post treatment, except for a combination of water-based and land-based AE, total duration of AE >2,000 minutes, frequency of training 1 or >3 times/week and intensity <50% maxHR and attention placebo as control. Statistical heterogeneity of analysis for the effect size for pain was substantially increased in the case of a total duration of AE <1,000 minutes and attention placebo as control (see Table 4).

#### Sensitivity analysis

Sensitivity analyses according to potential risks of bias for the outcome pain at post treatment did not change the significant effect of AE on pain at post treatment, except for studies with sample size <25 and with only median of outcomes available. Statistical heterogeneity of analysis for the effect size for pain was substantially increased in the case of studies that included patients with mental disorders and with only the median of outcomes available (see Table 5).

## Discussion

### Summary of evidence

AE reduces pain, fatigue and depressed mood, and improves HRQOL and physical fitness, at post treatment. Positive effects of AE on depressed mood, HRQOL and physical fitness can be detected at latest follow-up. AE has no positive effect on sleep at post treatment, and on pain, fatigue and sleep at follow-up. Continuing exercise is necessary to maintain positive effects on pain.

The following statements are valid for pain reduction at post treatment. There is no evidence of a superiority of water-based over land-based exercise. AE with a slight to moderate intensity is effective. Low-intensity AE (<50% maxHR) is not effective. A frequency of AE of 2 to 3 times/week for at least 4 to 6 weeks is necessary for a reduction of symptoms. Combining AE with stretching or strengthening is no more effective than AE alone.

The evidence is applicable to the majority of patients in clinical practice except patients with internal and orthopaedic diseases that may prevent AE and male patients.

### Limitations

Although every effort was made to obtain missing data (outcomes, study design) from the trial authors, it was not possible in every case to obtain these data; the included studies are therefore not represented fully in the meta-analyses. Only medians were available for three studies, but excluding these studies from analysis did not change the results.

The exercise protocol was insufficiently reported by some trials. The positive effects of the training can therefore possibly be attributed to other forms of exercise such as strength, stretching or relaxation, or in the case of pool-based exercise to the effects of warm water. Subgroup analyses did not, however, show a superiority of mixed exercise versus aerobic exercise nor a superiority of pool-based exercise versus land-based exercise.

The prescribed training intensity was either not assessed by heart rate telemetry or was not reported. No definitive conclusions on an effective intensity of AE are therefore possible.

The attendance rates during the study were inconsistently reported. If continuation of exercise until follow-up was recommended was inconsistently reported too. A subgroup analysis of studies with and without recommended exercise at follow-up was thus not possible.

Side effects were inconsistently reported. No definitive statement on the safety of AE in FMS is therefore possible.

The methodological quality of the studies varied. The positive effect on pain, however, was robust against potential methodological biases.

Given that formal blinding of participants and clinicians to the treatment arm is not possible in trials of exercise, we could have underestimated the extent to which clinicians' and participants' knowledge of group assignation influenced the true effect.

Males and adolescents were rarely included in the study populations. As no gender comparisons were reported, the evidence for the efficacy of AE in men and adolescents with FMS is limited.

#### Agreements and disagreements with other systematic reviews

Our meta-analysis does not confirm the conclusion of a qualitative systematic review that the greatest effects occurred in exercise programmes that were of lower intensity than those of higher intensity [13]. Our data that AE reduces pain at post treatment are in line with the conclusion of the meta-analyses of the Ottawa Panel [13] and are in contrast to that of the Cochrane review [12]. Not only moderate-intensity AE as recommended by the American Pain Society [5], but also low-intensity AE seems to be effective in reducing pain.

## Conclusions

### Implications for clinical practice

The amount and intensity of initial AE should be adapted to the individual level of physical fitness. Patients should start at levels just below their capacity and gradually increase the duration and intensity until they are exercising with low to moderate intensity for 20 to 30 minutes 2 to 3 times/week [12]. It does not seem necessary to assess the heart rate during AE to find the optimum intensity. Patients should exercise with an intensity at which they are able to speak fluently with another person [17]. The choice of the type of AE should be left to the patient's preferences and comorbidities and to the local offers of AE [11]. A training programme should last a minimum of 4 weeks. Patients should be educated that they may have some tolerable short-term increases in pain and fatigue but, if they exercise at an appropriate intensity, these symptoms should return to baseline levels within the first few weeks of exercise [12,17]. Patients should be motivated to continue exercise if they perceive a reduction of symptoms after the programme.

Because AE does not reduce sleeping disturbances, a combination of AE with medication effective for improving sleep - for example, tricyclic or dual antidepressants or pregabalin [81,82] - should be considered.

### Implications for research

Four main questions need to be answered by future studies. By which methods (for example, education, booster sessions) can patients be motivated to continue exercise? Is aerobic and mixed exercise cost-effective [83]? Is the combination of AE with pharmacological therapy superior to AE or medication alone? Which sociodemographic and clinical variables predict a positive and negative treatment outcome?

Future studies on these topics should focus on larger sample sizes (multicentre studies including a sufficient number of men and adolescents and patients with mental and somatic comorbidities). Study quality could be improved by detailed reporting of demographic and clinical data of the study groups at baseline, exercise protocol and adherence to interventions (attendance rates, adherence to prescribed intensity assessed by heart rate telemetry), creation of a standardised protocol to report adverse events and use of standard outcome measures.

## Abbreviations

AE: aerobic and mixed exercise; FMS: fibromyalgia syndrome; HRQOL: health-related quality of life; maxHR: maximum heart rate; RCT: randomised controlled trial; SD: standard deviation; SMD: standardised mean difference.

## Competing interests

WH received honoraria for educational lectures from Eli Lilly, Janssen-Cilag and Mundipharma, consulting fees from Eli-Lilly and Pfizer, and a congress travel grant from Eli-Lilly. MSc received consulting honoraria from Pfizer and MSD. None of these organisations financed this manuscript (including the article-processing charge). The other authors declare that they have no competing interests.

## Authors' contributions

WH conceived the hypothesis of the manuscript, participated in the data collection, conducted the statistical analysis, wrote the first draft of the manuscript and had primary responsibility for the manuscript. PK, JL, BM, MSt, MSc and AB participated in the collection of the data and analysis of the studies (see Materials and methods). AB and MSc participated in the study design and the interpretation of the data. All authors critically reviewed, contributed and approved the final manuscript.

## Authors' information

WH and MSc are vice-presidents of the German Interdisciplinary Association of Pain Therapy DIVS and were responsible for the development on the German interdisciplinary guideline on the classification, pathophysiology and management of FMS. AB is head of the Cochrane group on fibromyalgia.

## Supplementary Material

Additional file 1 **Search strategy for MEDLINE**. The file contains the literature search strategy for the database MEDLINE. Click here for file

Additional file 2 **Main characteristics of studies with aerobic and mixed exercise in fibromyalgia syndrome**. The file contains the main characteristics of studies with aerobic and mixed exercise in fibromyalgia syndrome including outcomes measures. Click here for file

Additional file 3 **Main characteristics of studies with head to head comparisons of different types of aerobic and mixed exercise in fibromyalgia syndrome**. The file contains the main characteristics of studies with head-to-head comparisons of different types of aerobic and mixed exercise in fibromyalgia syndrome including outcome measures. Click here for file

Additional file 4 **Effect estimates (standardised mean differences) of aerobic exercise versus controls on pain at post treatment**. Forest plots show standardised mean differences (effect sizes) from the random effects model (inverse variance method). A negative effect indicates that the endpoint score of the outcome in the exercise groups is lower than in control group in the study. The pooled (all studies together) effect size is weighted by the inverse variance of each study. IV, inverse variance (method); SD, standard deviation; Std. mean difference, standardised mean differences; random, random effects model; SD, standard deviation; total, number of patients; weight, relative weight (%) of the study in the calculation. Click here for file

Additional file 5 **Effect estimates (standardised mean differences) of aerobic exercise versus controls on fatigue and sleep at post treatment**. Forest plots show standardised mean differences (effect sizes) from the random effects model (inverse variance method). A negative effect indicates that the endpoint score of the outcome in the exercise groups is lower than in control group in the study. The pooled (all studies together) effect size is weighted by the inverse variance of each study. IV, inverse variance (method); SD, standard deviation; Std. mean difference, standardised mean differences; random, random effects model; SD, standard deviation; total, number of patients; weight, relative weight (%) of the study in the calculation. Click here for file

Additional file 6 **Effect estimates (standardised mean differences) of aerobic exercise versus controls on depressed mood at post treatment**. Forest plots show standardised mean differences (effect sizes) from the random effects model (inverse variance method). A negative effect indicates that the endpoint score of the outcome in the exercise groups is lower than in control group in the study. The pooled (all studies together) effect size is weighted by the inverse variance of each study. IV, inverse variance (method); SD, standard deviation; Std. mean difference, standardised mean differences; random, random effects model; SD, standard deviation; total, number of patients; weight, relative weight (%) of the study in the calculation. Click here for file

Additional file 7 **Effect estimates (standardised mean differences) of aerobic exercise versus controls on quality of life at post treatment**. Forest plots show standardised mean differences (effect sizes) from the random effects model (inverse variance method). A negative effect indicates that the endpoint score of the outcome in the exercise groups is lower than in control group in the study. The pooled (all studies together) effect size is weighted by the inverse variance of each study. IV, inverse variance (method); SD, standard deviation; Std. mean difference, standardised mean differences; random, random effects model; SD, standard deviation; total, number of patients; weight, relative weight (%) of the study in the calculation. Click here for file

Additional file 8 **Effect estimates (standardised mean differences) of aerobic exercise versus controls on physical fitness at post treatment**. Forest plots show standardised mean differences (effect sizes) from the random effects model (inverse variance method). A negative effect indicates that the endpoint score of the outcome in the exercise groups is lower than in control group in the study. The pooled (all studies together) effect size is weighted by the inverse variance of each study. IV, inverse variance (method); SD, standard deviation; Std. mean difference, standardised mean differences; random, random effects model; SD, standard deviation; total, number of patients; weight, relative weight (%) of the study in the calculation. Click here for file

Additional file 9 **Effect estimates (standardised mean differences) of aerobic exercise versus controls on pain and fatigue at latest follow-up**. Forest plots show standardised mean differences (effect sizes) from the random effects model (inverse variance method). A negative effect indicates that the endpoint score of the outcome in the exercise groups is lower than in control group in the study. The pooled (all studies together) effect size is weighted by the inverse variance of each study. IV, inverse variance (method); SD, standard deviation; Std. mean difference, standardised mean differences; random, random effects model; SD, standard deviation; total, number of patients; weight, relative weight (%) of the study in the calculation. Click here for file

Additional file 10 **Effect estimates (standardised mean differences) of aerobic exercise versus controls on sleep and depressed mood at latest follow-up**. Forest plots show standardised mean differences (effect sizes) from the random effects model (inverse variance method). A negative effect indicates that the endpoint score of the outcome in the exercise groups is lower than in control group in the study. The pooled (all studies together) effect size is weighted by the inverse variance of each study. IV, inverse variance (method); SD, standard deviation; Std. mean difference, standardised mean differences; random, random effects model; SD, standard deviation; total, number of patients; weight, relative weight (%) of the study in the calculation. Click here for file

Additional file 11 **Effect estimates (standardised mean differences) of aerobic exercise versus controls on quality of life and physical fitness at latest follow-up**. Forest plots show standardised mean differences (effect sizes) from the random effects model (inverse variance method). A negative effect indicates that the endpoint score of the outcome in the exercise groups is lower than in control group in the study. The pooled (all studies together) effect size is weighted by the inverse variance of each study. IV, inverse variance (method); SD, standard deviation; Std. mean difference, standardised mean differences; random, random effects model; SD, standard deviation; total, number of patients; weight, relative weight (%) of the study in the calculation. Click here for file

Additional file 12 **Effect estimates (standardised mean differences) of moderate versus low intensity on pain and depressed mood post treatment**. Forest plots show standardised mean differences (effect sizes) from the random effects model (inverse variance method). A negative effect indicates that the endpoint score of the outcome in the exercise group with moderate intensity is lower than in exercise group with low intensity. The pooled (all studies together) effect size is weighted by the inverse variance of each study. IV, inverse variance (method); SD, standard deviation; Std. mean difference, standardised mean differences; random, random effects model; SD, standard deviation; total, number of patients; weight, relative weight (%) of the study in the calculation. Click here for file

Additional file 13 **Effect estimates (standardised mean differences) of water versus land-based aerobic exercise on pain and depressed mood post treatment**. Forest plots show standardised mean differences (effect sizes) from the random effects model (inverse variance method). A negative effect indicates that the endpoint score of the outcome in the water-based exercise groups is lower than in the land-based exercise group in the study. The pooled (all studies together) effect size is weighted by the inverse variance of each study. IV, inverse variance (method); SD, standard deviation; Std. mean difference, standardised mean differences; random, random effects model; SD, standard deviation; total, number of patients; weight, relative weight (%) of the study in the calculation. Click here for file

Additional file 14 **Funnel plot of the comparisons of aerobic exercise versus controls on pain**. Scatter plot of the intervention effect estimates (standardised mean differences (SMD)) from individual studies against their standard errors (SE) (on a reversed scale). Publication bias may lead to asymmetry in funnel plots on visual inspection. Click here for file
