# Supplementary material for: Efficacy of different types of aerobic exercise in fibromyalgia syndrome: a systematic review and meta-analysis of randomised controlled trials
Source: Arthritis Res Ther. 2010 May 10;12(3):R79. doi: 10.1186/ar3002 (PMC2911859; doi:10.1186/ar3002)
Supplement: Additional file 1 — Search strategy for MEDLINE. The file contains the literature search strategy for the database MEDLINE. [file ar3002-S1.doc]

Additional file 1: Search strategy for Medline

1. exp Fibromyalgia
2. fibromyalgia.tw.
3. fibrositis.tw.
4. or/1-3
5. exp exercise
6. exp exertion
7. exp physical fitness
8. exp exercise test
9. exp exercise tolerance
10. exp sports
11. exp pliability
12. exp physical endurance
13. exertion$.tw.
14. exercis$.tw.
15. sport$.tw.
16. ((physical or motion) adj5 (fitness or therapy or therapies)).tw.
17. (physical$ adj2 edur$).tw.
18. manipulat$.tw.
19. (skate$ or skating).tw.
20. jog$.tw.
21. swim$.tw.
22. bicycl$.tw.
23. (cycle$ or cycling).tw.
24. walk$.tw.
25. (row or rows or rowing).tw.
26. aqua therapy.tw.
27. water therapy.tw.
28. spa therapy.tw.
29. pool therapy.tw.
30. or/5-29
31. 4 and 30
32. limit 31 to randomized controlled trial
