# Supplementary material for: Efficacy of different types of aerobic exercise in fibromyalgia syndrome: a systematic review and meta-analysis of randomised controlled trials
Source: Arthritis Res Ther. 2010 May 10;12(3):R79. doi: 10.1186/ar3002 (PMC2911859; doi:10.1186/ar3002)
Supplement: Additional file 2 — Main characteristics of studies with aerobic and mixed exercise in fibromyalgia syndrome. The file contains the main characteristics of studies with aerobic and mixed exercise in fibromyalgia syndrome including outcomes measures. [file ar3002-S2.doc]

Table 1: Main characteristics of studies with aerobic and mixed exercise in fibromyalgia syndrome

|  | Aerobic exercise (land- or water-based) versus controls | | | | | | | | | | | | | | |  |
| --- | --- | --- | --- | --- | --- | --- | --- | --- | --- | --- | --- | --- | --- | --- | --- | --- |
| Author  Country  Year  Setting  Referral  Reference Number | | Mean age  Women %  Race % | Exclusion criteria | Diagno-sis | STUDY POPULATION | | | TREATMENT GROUP | | | | Control group | Comedication allowed  Other cotherapies reported  Attendance rate  Side effects in AE group  Latest follow –up  Data on exercise at follow-up | Outcomes used for meta-analysis | | |
|  |  |  |  | Comorbi-dities assessed and reported | | N scree­ned/  Rando-mized (%) | N/comp­leting (%) | | N/completing (%) | Type of aerobic exercise  Length and intensity of aerobic exercise  Other type of exercise  Duration of total exercise | Kind of treatment  Duration treatment  N/completing (%) | |  | |  | |
| Alentorn-Geli  Spain  2008  University  Family physicians and local self-help groups  (53) | | 56 yrs  100% w  NR | Internal or orthopaedic diseases precluding exercise | ACR  Yes | | 104/36  (34.6) | 36/33  (91.7) | | 12/12  (100) | Group supervised  walking  30 min; 65-85% max HR  25 min stretching  2x60 min; 6 wks | Therapy as usual  12/10 (96) | | Any kind of pharmacological therapy allowed  NR  92%  NR  No follow-up | | Pain VAS 0-10  Fatigue VAS 0-10  Sleep NA  Depression VAS 0-10  HRQOL FIQ total  Phyfit NA | |
| Altan  Turkey  2004  University  NR  (54) | | 44 yrs  100% w  NR | Internal diseases  Mental disorder affecting compliance | ACR  NR | | NR | 50/46  (92) | | 25/24  (96) | Group  supervised  pool and out-of-pool-exercises  25 min; 60-75% max HR *  Min NR stretching  3X week for 35 min; 12 wks | Active control: Non-supervised  therapeutic pool without activities  3X week for 35 min; 12 wks  25/22  (88) | | Antidepressant and sedative drugs allowed, NSAID’s not allowed  NR  NR  No  12 weeks  NR | | Pain VAS 0-4  Fatigue VAS 0-4  Sleep VAS 0-3  Depression : BDI  HRQOL  FIQ total score  Phyfit NI | |
| Bircan  Turkey  2008  University  Referral outpatient clinics  (55) | | 48 yrs  100%  NR | Internal diseases | ACR  Yes | | NR | 30/26  (86.7) | | 15/13  (86.7) | Group supervised  treadmill training  20-30 min; 60-70% max HR  10 min stretching  3x40 min for 8 wks | Active control: Muscle strengthening  3x40 min for 8 wks  15/13  (86.7) | | Medication allowed and stable during study  NR  NR  NR  No follow-up | | Pain VAS 0-10  Fatigue VAS 0-10  Sleep VAS 0-10  Depression HADS  HRQOL NI  Phyfit 6-min walk test | |
| Buckelew  USA  1998  University  Referral physicians  (56) | | 44 yrs  90%  NR | Internal diseases  Psychosis | Yunus  NR | | 916/119  (13.0) | 119/109  (91.6) | | 30/28  (93.3) | Group supervised  walking  min NR; 60-70% submax HR  Min NR strength  1x1,5-3 h for 6 wks; home 2/wks recommen-ded  maintenance 1xmonth for 2 yrs | Attention control: education; dosage NR  30/28 (93.3)  Biofeedback and combination walking plus biofeedback not used for comparison | | Medication allowed; changes analysed for confounding outcomes  NR  NR  NR  104 weeks  Continuing exercise part of program | | Pain VAS 0-10  Fatigue NA  Sleep NA  Depression CES-D  HRQOL NA  Phyfit NA | |
| DaCosta  Canada  2005  University  Hospital and commun-ity rheumatologists and news-paper advertisement  (57) | | 49 yrs  100%  NR | Internal or other diseases preventing exercise | ACR  NR | | 105/80  (76.2) | 80/69  (86.2) | | 39/33  (84.6) | Single home-based  Land-based exercise details NR  Duration individuali-zed; 60-70% max HR  Stretching and strength individual-ized; min NR  1X60-120 min/wk for 12 wks | Usual care  40/36 (90) | | NR  NR  67%  NR  26 weeks  NR | | Pain VAS 0-10 NP  Fatigue NP  Sleep NP  Depression NP  HRQOL FIQ total  Phyfit NA | |
| Etnier  USA  2009  University  Local rheumatologists and news-paper advertisement  (58) | | 55 yrs  100%  NR | Internals diseases | ACR  NR | | 25/16  (80) | 16/16  (100) | | 8/8  (100) | NR  Supervised walking  30 min; 55-65% max HR  Min NR Stretching and strength;  3x60 min for 18 wks | Therapy as usual with delayed exercise treatment  8/8 (100) | | NR  NR  65%  NR  No follow-up | | Pain VAS 0-10 NP  Fatigue VAS 0-10  Sleep VAS 0-10 NP  Depression CES-D  HRQOL FIQ total  Phyfit | |
| Fontaine  USA  2007  University  Advertisements in newspapers and local FMS groups  (59) | | 50 yrs  94% w  91% white | Internal diseases  Intention to seek treatment for mental disorder during study | ACR  NR | | NR | 48/34  (70.8) | | 22/16  (72.7) | Single non-supervised walking  Walking up to 30 min; moderate intensity, details NR NR  None  5 to 7 days a week; 12 wks | Active control: Education 3 meetings  26/18 (69.2) | | NR  NR  NR  NR  No follow-up | | Pain VAS 0-10  Fatigue FSS  Sleep NA  Depression VAS 0-10  HRQOL FIQ total  Phyfit 6 min walk | |
| Gowans  Canada  2001  University  Advertisement in rheumatological ambula-tory depart-ment and self-help groups  (60) | | 45 yrs  89% w  NR | Internal diseases  Intended change in medication for mental disorders | ACR  Yes | | NR | 57/31 (54.3) | | 30/15  (50) | Group supervised  land- and pool-based  20 min; 60-75% max HR  10 min stretching  3xweek 30 min, 23 wks | Therapy as usual  27/16 (59.2) | | Pharmacological cotherapies allowed and controlled for;  exclusion if psychological therapy was initiated  67%  NR  52 weeks AE group only (25) | | Pain NI  Fatigue NA  Sleep NA  Depression  BDI  HRQOL FIQ total  PhyFit 6 min walk test | |
| Gusi  Spain  2006  University  Local self-help group  (61) | | 51 yrs  100% w  NR | Internal diseases  Disorder of the spine  Mental disorder | ACR  No | | 59/35  (59.3) | 35/34  (97.1) | | 18/17  (94.4) | Group  supervised pool exercise  20 min; 65-75% max HR  20 min stretching and strength  3xweek for 60 min; 12 wks | Therapy as usual  17/17 (100%) | | Cotherapies with antidepressants, analgesics, muscle relaxants allowed  No other psychologcial or physical therapy allowed  94% of the patients attend at least 95% of the sessions  NR  No exercise recommended until evaluation at follow-up | | Pain VAS 0-100  Fatigue NP  Sleep NA  Depression EQ 5-D  HRQOL FIQ total  Phyfit NI | |
| Jones  USA  2008  University  University  (62) | | 49 yrs  97% w  93% cau | Internal diseases  Unresolved ligitation  Major depression | ACR  NR | | NR | 226/207  (91.2) | | 47/39  (83) | Group  supervised aerobic exercise plus placebo  30 min; 40-50% max HR  10 min strength, 5 min flexibility, 5 min balance  3xweek for 60 min, 26 wks | Attention control: 1xweek telephone call; 1xmonth 2h visit  54/39  (72.2)  Study arms aerobic exercise plus pyrodostigmine and pyrodostigmine only not used for comparison | | NR  NR  NR  NR  No follow-up | | Pain VAS 0-10  Fatigue VAS 0-10  Sleep VAS 0-10  Depression VAS 0-10  HRQOL FIQ total  Phyfit Oxygen uptake | |
| King  Canada  2002  University  Rheumatologists or self-referred  (63) | | 45  100%w  NR | Internal diseases | ACR  Yes | | 259/152  (58.9) | 152/96  (63.1) | | 42/30  (71.4) | Group  supervised land- and pool-based exercise (walking, aquasize)  20-40 min; 60-75% max HR  Min NR stretching  3xweek 20-40 min; 12 wks | Attention control: Infomations on coping and stretching  34/18  (52.9)  Study arm education plus  Aerobic exercise not used for comparison | | Pharmacological therapies allowed  Psychological or alternative treatments controlled  NR  NR  12 weeks  NR | | Pain NP  Fatigue NP  Sleep VAS NP  Depression NP  HRQOL FIQ total  Phyfit 6 min walk test | |
| Martin  Canada  1996  University  Family practitio-ners, rheumato-logists and self-help organisations  (64) | | 44 yrs  95 w  NR | Internal diseases  Medication that affect normal response to exercise | ACR  No | | 68/60  (88.2) | 60/38  (63.3) | | 28/18  (64.2) | Group  supervised walking  20 min; 60-80% max HR  20 min flexibility and strength  3xweek 60 min; 6 wks | Active control: Supervised relaxation  3xweek for 60 min; 6 wks  2/20 (62.5) | | NR  No medication that could affect normal physiological response to exercise allowed  NR  NR  No follow-up | | Pain NP  Fatigue NP  Sleep NP  Depression NP  HRQOL FIQ total  Phyfit Treadmilll score | |
| Mc Cain  Canada  1988  University  Referrals to outpatient rheumatology depart-ment  (65) | | 38 yrs  NR  NR | Internal diseases | Smythe  No | | NR | 42/38  (90.5) | | 21/18  (85.7) | Group supervised  bicycle ergometer  50 min;  > 150 beats/min for gradually increasing time periods  None  3xweek 60 min, 20 wks | Active control:  Supervised strectching  3xweek for 60 min, 20 wks  21/20  (95) | | NR  Only acetaminophen allowed and outcomes controlled for  90%  NR  No follow-up | | Pain VAS 0-100  Fatigue NA  Sleep Hours of disturbed sleep per night  Depression NA  HRQOL NA  Phyfit Physical working capacity | |
| Mengs-hoel  Norway  1992  City hospital  Hospital and self-help groups  (66) | | 33 yrs  100% w  NR | Abnormal laboratory tests | ACR  NR | | NR | 35/25  (71.4) | | 18/11  (61.1) | Group  supervised aerobic dance  60 min; 120-150/min  None  2xweek 60 in; 20 wks | Therapy as usual  17/14 (82.3) | | NR  NR  NR  NR  No follow-up | | Pain VAS 0-100 **  Fatigue NA  Sleep VAS 0-100 **  Depression NA  HRQOL NA  Phyfit NI | |
| Munguia-Izquierdo  Spain  2008  University  Local self help organisation  (67) | | 50 yrs  100% w  100% white | Internal and mental diseases | ACR  NR | | 250/60  (24) | 60/53  (88.3) | | 35/29  (82.9) | Group  supervised aquatic exercise  20-30 min; 50-80% max HR  10-20 min strength  3xweek for 60 min; 16 wks | Usual medical care  25/24  (96) | | Regular medication allowed  No other psychologcial or physical therapy allowed;  88%  No side effects  No follow-up | | Pain NP  Fatigue NP  Sleep NP  Depression NP  HRQOL FIQ total score  Phyfit NI | |
| Nichols  USA  1994  University  Local self help organisation  (68) | | 48 yrs  89 %w  NR | Internal or orthopedic diseases preventing from exercise | ACR  NR | | NR | 24/19  (79.2) | | 12/10  (83.3) | Group  supervised  walking  20 min; 60-70% max HR  min NR stretching  3xwek 40 min, 8 wks | Usual medical care  12/9  (75) | | Medication not allowed  NR  NR  NR  No follow-up | | Pain rating index MPQ  Fatigue NA  Sleep NA  Depresion NA  HRQOL: NI  Phyfit NA | |
| Noregaard  Denmark  1996  University  Clinic register  (69) | | 44 yrs  NR  NR | Internal diseases  Alcoholism | ACR  NR | | 218/38  (17.4) | 38/23  (60.5) | | 15/5  (33.3) | Group  supervised  aerobic dancing  40 min; 40-50% max HR  None  3xweek 50 min; 12 wks | Active control:  Hot packs 2x30 min 12 wks  8/7(87.5)  Study arm body awareness combined with stretching not used for comparison | | Stable medication allowed  NR  NR  NR  No follow-up | | Pain VAS 0-10 **  Fatigue VAS 0-10 **  Sleep VAS 0-10 **  Depression BDI **  HRQOL FIQ total **  Phyfit Physical working capacity ** | |
| Ramsay  UK  2000  Regional hospital  NR  (70) | | NR  NR  NR | NR | ACR  NR | | NR | 74/NR | | NR | Group  supervised jogging on the spot  Intensity NR;  Min NR;  Min NR stretching  1xweek 60 min; 12 wks | Attention control:  Single session demonstration of AE, stretching and relaxation  NR | | Stable medication with tricyclics, NSAID’s and analgesics  NR  40% attented at least 75% of the classes  NR  36 weeks  Written advice to continue exercise | | Pain VAS 0-100 **  Fatigue NA  Sleep NA  Depression HADS **  HRQOL NA  Phyfit NI | |
| Redondo  Spain  2004  University  General practitio-  Ners  (71) | | NR  100% w  NR | Serious concomitant diseases | ACR  NR | | 56/40  (71.4) | 40/31  (77.5) | | 19/15  (78.9) | Group  supervised  pool exercises, cycle ergo-meter and isokinetic exercises  min NR; 50-80% max HR *  Min NR flexibility and strength  5xweek 45 min; 8 wks | Active control:  CBT 1x2,5 h/week for 8 wks  21/16 (76.2) | | Flexible medication with NSAID’s, amitriptyline and acetaminophen allowed  NR  84%  NR  52 Weeks  Instruction to maintain exercise | | Pain VAS 0-10  Fatigue VAS 0-10  Sleep VAS 0-10  Depression BDI HRQOL FIQ total  Phyfit Oxygen uptake | |
| Richards  GB  2002  City Hospital Rheumatology Clinic  Clinic register  (72) | | 48  92% w  NR | Severe somatic disorder | ACR  No | | 196/136  (69.4) | 136/115  (84) | | 69/58  (84) | Group  supervised cycle ergometer  Intensity as tolerated (perspire slightly while still able to talk in complete sentences)  ; 25 min  None  2Xweek 60 min, 12 wks | Active control:  Supervised relaxation training  2Xweek 60 min, 12 wks  67/57 (85.1) | | NR  NR  27%  None  40 weeks  NR | | Pain NP  Fatigue NP  Sleep NP  Depression NP  HRQOL FIQ total  Phyfit NA | |
| Rooks  USA  2007  Commu-nity and hospital fitness center  Rheuma-tologists  (73) | | 48  100%  83% w | Medical conditions that limited ability to perform exercise | ACR  Yes | | 356/207  (58.1) | 207/135  (65.2) | | 51/35  (68.7) | Group  supervised walking or cycle ergometer  Self determined level of moderate effort; up to 45 min  None  2xweek, 60min, 16 wks | Active control:  Education program  7X 120 min  55/38 (69)  Study arms Strength and combination strength with education not used for comparison | | NR  NR  73%  None  No follow-up | | Pain VAS 0-10  Fatigue VAS 0-10  Sleep NA  Depressed mood BDI  HRQOL FIQ total  Phyfit 6 min walk | |
| Schachter  Canada  2003  University  Rheumatologists, family physician and advertisement  (74) | | 42 yrs  100% w  94% cau | Severe somatic disease | ACR  NR | | NR | 143/102  (71.3) | | a. 51/36  (70.8)  b. 56/35  (62.5) | Group  supervised rhythmic movements  40-75% max HR  None  a. 2 sessions/day up to 15 min  b. Once a day up to 30 min each 16 wks | Attention control:  Monthly group meetings without education  36/31 (86.1) | | No control for medication  No new non-pharmacological treatments  Appr 50%  Increase of pain and fatigue, % NR  No follow-up | | Pain VAS 0-10  Fatigue VAS 0-10  Sleep NA  Depression VAS 0-10  HRQOL FIQ total  Phyfit Oxygen uptake | |
| Sencan  Turkey  2004  University  NR  (75) | | 34.5 yrs  100 % w  NR | No other comorbid diasease  Drug dependency | ACR  No comorbi-dities | | 67/60  (89.5) | 60/60  (100) | | 20/20  (100) | Group  supervised cycle ergometry *  30 min, intensity NR  None  3xweek 40min, 6 wks | Attention control:  TENS-Placebo 3x20 min 6 wks  20/20 (100)  Study arm 20 mg/paroxetin/d not used for comparison | | Paracetamol allowed  No other medication allowed  NR  NR  20 weeks  Motivation to continue exercise | | Pain VAS 0-10  Fatigue NA  Sleep NA  Depression BDI  HRQOL NA  Phyift NA | |
| Tomas-Carus  Spain  2008  University  Local self-help organisation  (76) | | 50 yrs  100% w  NR | Internal diseases  Severe psychiatric disorder | ACR  NR | | 59/34  (57.6) | 34/33  (97) | | 17/16  (94) | Group  supervised aquatic exercise  20 min; 65-75% of max HR  20 min flexibility and strength  3week for 60 min, 12 wks | Therapy as usual: Usual daily activities  15/14  (93.3) | | NR  No other psychologcical or physical therapies allowed  NR  NR  No follow-up | | Pain VAS 0-10  Fatigue VAS 0-10  Sleep NA  Depression VAS 0-10  HRQOL FIQ total  Phyfit Oxygen uptake | |
| Valim  Brazil  2002  University  Rheumatology outpatient clinic  (77) | | 46 yrs  100% w  NR | Internal diseases | ACR  NR | | NR | 76/60  (78.9) | | 38/32  (84.2) | Group  supervised  Walking  35-40 min; 60-65% max HR  None  3xweek for 45 min x20 wks | Active control:  Supervised streching  3xweek for 45 min x20 wks  38/28  (73.6) | | Acetaminophen as rescue  No other therapy  NR  NR  No follow-up | | Pain VAS 0-10  Fatigue NP  Sleep NP  Depression BDI  HRQOL FIQ total  Phyfit Oxygen uptake | |
| Valkeinen  2008  Finland  University  Outpatient clinic  (78) | | 59 yrs  100% w  Yes | Internal diseases  Other diseases that might confound study results | ACR  NR | | 180/26  (14.4) | 26/24  (92.3) | | 15/13  (86.7) | Group  supervised and non-supervised walking or cycling  Min NR; 40-80% max HR  None  2xweek for 60 minx21 wks | Active control:  Supervised stretching  2xweek 60 minX21 wks  11/11  (100) | | Previous medication allowed  NR  NR  NR  No follow up | | Pain VAS 0-100  Fatigue 0-100  Sleep 0-100  Depression: NA  HRQL : VAS 0-100  Phyfit : Oxygen uptake | |
| Van Santen  Nether-lands  2002  University  Central Registry of rheumatic diseases  (79) | | 43 yrs  100 % w  NR | Internal diseases  Incapacita-ting psychology-cal distress  Unsettled disability reviews | ACR  NR | | 168/143  (85.1) | 143/118  (82.5) | | 58/47  (81) | Group  supervised land-based exercise, details NR;  30 min; Intensity left up to patient (no more than moderate intensity performed)  10 min strength-ening  2xweek for 60min, 24 wks | Usual care  29/28 (96.5)  Study arm biofeedback group not used for comparison | | NR  NR  64% >66% participation  NR  No follow-up | | Pain VAS 0-10  Fatigue VAS 0-100  Sleep NA  Depression NA  HRQOL SIP total  Phyfit Wattmax | |
| Wigers  1996  Norway  University  Local patient associations and Outpatient depart-ment  (80) | | 43 yrs  90% w  NR | NR | ACR  NR | | 76/60  (78.9) | 60/48  (80%) | | 20/16  (80) | Group  supervised  Land-based games  45 min; 60-70% max HR  None  3x45 min for 14 wks | Usual care  20/17 (85)  Study arm stress management not used for comparison | | Baseline treatment unchanged  Exclusion form analysis if new therapies were initiated  37.5%  1/20 drop out due to ischialgia  208 weeks; 4/16 continued exercise; continuers with less pain, depression and improved Phyfit compared to non-continuers | | Pain VAS 0-10  Fatigue VAS 0-10  Sleep VAS 0-10  Depression VAS 0-10  HRQOL NA  Phyfit Physical Working capacity | |

Abbreviations and symbols

* Data given to the Cochrane group on request; ** only median available for analysis

BDI= Beck Depression Inventory; CDI= Children’s Depression Index; CES-D = Center for Epidemiologic Studies Depression Scale; EQ5-D= Europe Quality of Life Dimension FIQ= Fibromyalgia Impact Questionnaire; FSS= Fatigue Severity Scale; HADS= Hospital Anxiety and Depression Scale; HRQOL= Health-related quality of life; max HR = maximal heart rate (220-age [years]; NA= Not assessed; NI= Outcome not meeting inclusion criteria; NP= Not reported and not provided on request; NSAID’s= Nonsteroidal agents; PSQI = Pittsburgh Sleep Quality Index; SIP= Sickness Impact Profile total score; VAS= Visual Analogue Scale;

Measures of physical fitness: Maximum or submaximal oxygen intake: ml/min/kg of body weight; Physical work capacity (at calculated 170 beats per minute): kilopond-meters; Six-minutes walk test: meters; Treadmill score: seconds until volitional exhaustion; Wattmax = Maximum watt at bicycle test

Note: The order of the presented studies is arranged according to alphabetic order
