# Supplementary material for: Efficacy of different types of aerobic exercise in fibromyalgia syndrome: a systematic review and meta-analysis of randomised controlled trials
Source: Arthritis Res Ther. 2010 May 10;12(3):R79. doi: 10.1186/ar3002 (PMC2911859; doi:10.1186/ar3002)
Supplement: Additional file 3 — Main characteristics of studies with head to head comparisons of different types of aerobic and mixed exercise in fibromyalgia syndrome. The file contains the main characteristics of studies with head-to-head comparisons of different types of aerobic and mixed exercise in fibromyalgia syndrome including outcome measures. [file ar3002-S3.doc]

Additional file 3: Main characteristics of studies with head to head comparisons of different types of aerobic and mixed exercise in fibromyalgia syndrome

|  | **Different intensities of aerobic exercise** | | | | | | | | | | | | | | | | | | | | | | | | |  |
| --- | --- | --- | --- | --- | --- | --- | --- | --- | --- | --- | --- | --- | --- | --- | --- | --- | --- | --- | --- | --- | --- | --- | --- | --- | --- | --- |
| Author  Country  Year  Setting  Referral  Reference  number | | Mean age  Women %  Race % | | Exclusion criteria | | Diagno-sis | | STUDY POPULATION | | | | | TREATMENT GROUP  High/ Moderate intensity training | | | | | | | TREATMENT GROUP  Low intensity training | | Comedication allowed  Other cotherapies reported  Attendance rate  Side effects in AE group  Latest follow-up  Data on exercise at follow-up | | Outcomes used for meta-analysis | | |
|  |  |  |  |  |  | Comorbi-dities assessed and reported | | | N scree­ned/  Rando-mized (%) | | N/comp­leting (%) | | | N/completing (%) | | Type of aerobic exercise  Length and intensity of aerobic exercise  Other type of exercise  Duration of total exercise | | Kind of treatment  Duration treatment  N/completing (%) | | | |  | | |  | |
| Meyer  2000  USA  University  Clinics, physicians, support groups  (46) | | 49 yrs  100 w | | Severe somatic diseases | | ACR | | | NR | | 21/8  (38.1) | | | 8/3 (37.5) | | Group  non-supervised walking  12 up to 30 min; 40 up to 85% max HR  None  3xweek, average 21 min, 24 wks | | Non-supervised walking  12 up to 30 min; 25 up to 60% max HR  None  3xweek, average 21 min, 24wks  8/5 (62.5) | | | | NR  NR  Low, details NR  Plantar fascitis, %  NR  No follow-up | | | Pain HAQ 0-3  Fatigue NA  Sleep NA  Depression NA  HRQOL FIQ total  Phyfit NR | |
| Stephens  USA  2008  District clinic  Rheumatology and Pain clinics of the same hospital  (48) | | 13.6 yrs  71.4 w  NR | | Severe somatic disorder  Unstable medication | | ACR  No | | | 537/30  (5.6) | | 30/24  (80) | | | 14/12  (85.7) | | Group supervised and single home-based cardio-dance and boxing movements  20 min; 70% max HR  None  3xweek 30 min, 12 wks  Yes | | Supervised and home-based Qi-Gong,  20 min, < 70% max HR  None  3xeek 30 min, 12 wks  16/12 (75) | | | | Stable medication  NR  64%  None  No follow-up | | | Pain VAS 0-10  Fatigue NI  Sleep NA  Depression CDI  HRQOL FIQ total  Phyfit Watt max | |
| Van Santen  2002  Nether  Lands  University  Central registry of rheumatic diseases  (49) | | 42 yrs  100 w | | Severe somatic disease  Incapacita-  Ting pychological distress  Disability compnesa-tion disputes | | ACR | | | 163/37  (22.7) | | 37/30  (81.8) | | | 19/17  (89.5) | | Group  supervised bicycle ergometer  45 min; 70% max HR  None  3xweek, 60 min, 20 wks | | Supervised bicyle ergometer,  30 min bicycle alternating with stretching, intensity left up to the patient  Min NR; Stretching and balance  2xweek, 60 min, 20 wks  16/13 (81.2) | | | | Baseline medication and  physiotherapy allowed  No  50% participation rate > 67%  NR  No follow-up | | | Pain VAS 0-100  Fatigue NA  Sleep NA  Depression SCL 90-R  HRQOL NA  Phyfit Watt max | |
|  | |  | |  | |  | | |  | |  | | |  | |  | |  | | | |  | | |  | |
|  | **Head- to head comparisons water vs. land -based aerobic exercise** | | | | | | | | | | | | | | | | | | | |  | | | | | |
| Assis  Brazil  2006  University  NR  (50) | | | 43 yrs  100% w  NR | | Internal diseases | | ACR  NR | | | 276/60  (21.7) | | 60/52  (86.7) | | | 30/26  (86.7) | | Group  supervised  Aquatic exercise pool ° NR  40 min,individual anaerobic threshold  No  3xweek for 60min for 15 wks | | Supervised  Walking or jogging  40 min, individual anaerobic threshold  No  3xweek for 60min for 15 wks  30/26 (86.7) | | | | Medication allowed and stable during study; rescue medication acetaminophen  NR  NR  NR  No follow-up | | Pain VAS 0-10  Fatigue VAS 0-10 *  Sleep VAS 0-10 *  Depression VAS 0-10 *  HRQOL FIQ total score  Phyfit NA  7 | |
| Evcik  Turkey  2008  NR  (51) | | | 42 yrs  100% w  NR | | Internal disease  Intake of antidepressants or NSAR’s | | ACR  NR | | | NR | | 63/61  (96,8) | | | 33/31  (93.9) | | Group  supervised  Aquatic exercise, pool 33°  35 min aquatic exercise; intensity NR  None  3xweek for 60 min; 5 wks | | Non supervised home-based aerobic  Min NR, intensity NR  Min NR, stretching  30/30  (100) | | | | NR  NR  NR  No side effects  19 weeks  NR | | Pain VAS 0-10  Fatigue NP  Sleep VAS 0-10 *  Depression BDI  HRQOL FIQ total  2 | |
| Jentoft  Norway  2001  University  NR  (52) | | | 41 yrs  100% w  NR | | Internal diseases | | ACR  NR | | | NR | | 44/34  (77.3) | | | 22/18  (81.8) | | Group  supervised aquatic exercise in 34°C pool  25 min aquatic training, 60-80% max HR  20 min strength  2xweek for 60 min; 20 wks | | Supervised exercise in gym hall  25 min aerobic dance, 60-80% max HR  20 min strength  2xweek for 60 min; 20 wks  22/16  (80) | | | | NR  NR  NR  NR  26 weeks  Encouraged to continue excercise | | Pain VAS 0-10  Fatigue VAS 0-10  Sleep VAS 0-10  Depression VAS 0-10  HRQOL FIQ total score  Phyfit Maximum oxygen uptake  4 | |
| Vitorino  Brazil  2006  University  NR  (53) | | | 47 yrs  100 w  NR | | NR | | ACR  NR | | | NR | | 50/47  (94) | | | 25/24  (96) | | Group  supervised aquatic exercise,  Pool °C NR  30 min, intensity NR  10 min stretching  3xweek for 60 min; 3 wks | | Supervised bicycle ergometer training  30 min, intensity NR  10 min stretching  3xweek for 60 min; 3 wks  25/23 (95) | | | | NR  NR  NR  NR  N follow up | | Pain SF-36  Fatigue NA  Sleep Total Nap time min  Depression NA  HRQOL NR  Phyfit NA  6 | |

Abbreviations and symbols

* Data given on request; ** only median available for analysis

BDI= Beck Depression Inventory; FIQ= Fibromyalgia Impact Questionnaire; HAQ = Health Assessment Questionnaire; HRQOL= Health-related quality of life; max HR = maximal heart rate (220-age [years]; NA= Not assessed; NI= Outcome not meeting inclusion criteria; NP= Not reported and not provided on request; Phyfit = Physical fitness; SCL 90-R = Symptom Checklist 90-R; VAS= Visual Analogue Scale;

Measures of physical fitness: Maximum or submaximal oxygen intake: ml/min/kg of body weight; Physical work capacity (at calculated 170 beats per minute): kilopond-meters; Six-minutes walk test: meters; Treadmill score: seconds until volitional exhaustion; Wattmax = Maximum watt at bicycle test

Note: The order of the presented studies is arranged according to alphabetic order
