# Supplementary material for: Efficacy of different types of aerobic exercise in fibromyalgia syndrome: a systematic review and meta-analysis of randomised controlled trials
Source: Arthritis Res Ther. 2010 May 10;12(3):R79. doi: 10.1186/ar3002 (PMC2911859; doi:10.1186/ar3002)
Supplement: Additional file 8 — Effect estimates (standardised mean differences) of aerobic exercise versus controls on physical fitness at post treatment. Forest plots show standardised mean differences (effect sizes) from the random effects model (inverse variance method). A negative effect indicates that the endpoint score of the outcome in the exercise groups is lower than in control group in the study. The pooled (all studies together) effect size is weighted by the inverse variance of each study. IV, inverse variance (method); SD, standard deviation; Std. mean difference, standardised mean differences; random, random effects model; SD, standard deviation; total, number of patients; weight, relative weight (%) of the study in the calculation. [file ar3002-S8.doc]

Additional file 8: Effect estimates (standardised mean differences) of aerobic exercise versus controls on physical fitness at posttreatment

**Physical fitness**
