# Supplementary material for: Efficacy of different types of aerobic exercise in fibromyalgia syndrome: a systematic review and meta-analysis of randomised controlled trials
Source: Arthritis Res Ther. 2010 May 10;12(3):R79. doi: 10.1186/ar3002 (PMC2911859; doi:10.1186/ar3002)
Supplement: Additional file 12 — Effect estimates (standardised mean differences) of moderate versus low intensity on pain and depressed mood post treatment. Forest plots show standardised mean differences (effect sizes) from the random effects model (inverse variance method). A negative effect indicates that the endpoint score of the outcome in the exercise group with moderate intensity is lower than in exercise group with low intensity. The pooled (all studies together) effect size is weighted by the inverse variance of each study. IV, inverse variance (method); SD, standard deviation; Std. mean difference, standardised mean differences; random, random effects model; SD, standard deviation; total, number of patients; weight, relative weight (%) of the study in the calculation. [file ar3002-S12.doc]

Additional file 12: Effect estimates (standardised mean differences) of moderate versus low intensity on pain and depressed mood posttreatment

**Pain**

**Depressed mood**
