# Supplementary material for: Efficacy of different types of aerobic exercise in fibromyalgia syndrome: a systematic review and meta-analysis of randomised controlled trials
Source: Arthritis Res Ther. 2010 May 10;12(3):R79. doi: 10.1186/ar3002 (PMC2911859; doi:10.1186/ar3002)
Supplement: Additional file 14 — Funnel plot of the comparisons of aerobic exercise versus controls on pain. Scatter plot of the intervention effect estimates (standardised mean differences (SMD)) from individual studies against their standard errors (SE) (on a reversed scale). Publication bias may lead to asymmetry in funnel plots on visual inspection. [file ar3002-S14.doc]

Additional file 14: Funnel plot of the comparisons of aerobic exercise versus controls on pain

**Pain**

Figure legend: Scatter plot of the intervention effect estimates (Standardised mean differences [SMD]) from individual studies against their standard errors (SE) (on a reversed scale). Publication bias may lead to asymmetry in funnel plots on visual inspection.
